# Novel and Sustainable Materials for the Separation of Lithium, Rubidium, and Cesium Ions from Aqueous Solutions in Adsorption Processes—A Review

**DOI:** 10.3390/ma17246158

**Published:** 2024-12-17

**Authors:** Małgorzata A. Kaczorowska

**Affiliations:** Faculty of Chemical Technology and Engineering, Bydgoszcz University of Science and Technology, Seminaryjna 3, 85-326 Bydgoszcz, Poland; malgorzata.kaczorowska@pbs.edu.pl

**Keywords:** metal recovery, selective separation, adsorption, lithium, cesium, rubidium, “green” methods

## Abstract

The growing demand for alkali metals (AMs), such as lithium, cesium, and rubidium, related to their wide application across various industries (e.g., electronics, medicine, aerospace, etc.) and the limited resources of their naturally occurring ores, has led to an increased interest in methods of their recovery from secondary sources (e.g., brines, wastewater, waste leachates). One of the dynamically developing research directions in the field of separation of AMs ions from various aqueous solutions is the search for novel, efficient, and “green” materials that could be used in adsorption processes, also on a larger industrial scale. This review concerns the latest achievements (mainly from 2023 to 2024) in the development of innovative adsorption materials (e.g., ion sieves, aluminum-based adsorbents, mineral adsorbents, composites, resins) for the separation of Li^+^, Cs^+^, and Rb^+^ ions from solutions, with particular emphasis on their most important advantages and limitations, as well as their potential impact on the environment.

## 1. Introduction

Interest in alkali metals (AMs), which are widely used in various fields due to their properties, is steadily increasing. In the case of the most abundant AMs in nature, i.e., sodium and potassium, which play various crucial roles in biological systems, ongoing research focuses on their potential use in modern energy storage technologies, in nanomaterials, and in new generation fertilizers intended to boost crop yields and reduce environmental pollution [1,2,3,4]. Less abundant lithium, used in the manufacture of grease, glass, and pharmaceuticals, currently plays a key role in the production of lithium-ion batteries (LIBs), applied in electronic devices and electric vehicles [5,6]. The intensive utilization of Li defined even as the “symbolic element of the current energy revolution” [6] in new technologies is closely related to research on the modification of various types of lithium-based batteries in order to improve their performance and stability and reduce their production costs [7,8]. Lithium is also being increasingly used in the production of aluminum-lithium alloys, characterized by appropriate density and flexibility suitable for applications in aircraft and space technology [9]. Rubidium, due to its properties (e.g., photochemical activity, thermal and electrical conductivity, biochemical catalytic properties, etc.), has been utilized in electronics, medicine, and aerospace, but it also plays a critical role in energy conversion (e.g., thermionic power generation) [10]. Various rubidium compounds, which can potentially be used, for example, in catalysis, are systematically synthesized and analyzed [11]. Cesium, which has been used in various fields (e.g., in catalysis, for the production of photoelectric cell components, in X-ray radiation for cancer treatments, or in the oil and gas industry), is also starting to play an increasingly important role in energy storage applications [12].

The extensive use of alkali metals in different areas leads to significant amounts of AM-containing waste and sewage (e.g., industrial, agricultural, municipal), which may pose a threat to the natural environment but at the same time may be exploited as a secondary source of valuable, critical elements. The potential threat to the environment associated with AMs is partly related to the introduction of significant amounts of sodium and potassium compounds (e.g., salts), originating from sewage/waste, to the soil/groundwater, which negatively affects plant development [13,14]. Therefore, research is being conducted to develop efficient, economical, and environmentally friendly methods for removing sodium and potassium ions from various aqueous solutions [15,16]. An example of solutions with high salt content (e.g., NaCl) are brines generated in large quantities as by-products in seawater desalination plants. When treating brines, it is important not only to remove elements posing a potential threat to the environment, such as high concentrations of Na^+^ ions (which have negligible economic recovery value) but also to explore the possibility of recovering critical elements present in low concentrations, such as Li and Rb ions, among others. Sustainable extraction of metals, including rare AMs from brines, is beneficial in the context of the European Green Deal (aiming to switch from fossil fuels to renewable energy sources, which is associated with increased use of critical raw materials) and helps reduce the exploitation of natural resources [17]. Additionally, desalinated water is becoming an increasingly important source of drinking water in many regions of the world due to the reduction in freshwater resources caused by such factors as climate change, environmental pollution, or population growth. The increased production of drinking water from seawater results in larger amounts of brine, leading to growing interest in developing methods for their appropriate treatment and sustainable recovery of valuable raw materials [18,19]. Regarding the possibility of recovering valuable alkali metals, a particularly important group of waste is used batteries, e.g., lithium-ion batteries, found in many electrical devices. Since in recent years there has been a steady increase in the number of used lithium-ion batteries generated (e.g., from electric cars), the development of efficient and safe methods of recycling them (and recovering valuable lithium) is necessary, both to limit the extraction of critical Li from primary sources and to protect the environment [20]. In the case of rubidium, intermediate products generated during the processing of minerals to extract Li and Cs (e.g., lepidolite, which is a mica mineral containing lithium) are currently the main sources from which Rb is recovered (through acid digestion or roasting and leaching processes), but research is also being carried out on the possibility of recovering this element from salt lakes, seawater, and brines that usually contain significant reserves of the element [21]. Many types of industrial waste generated in the processes of extraction of raw materials from ores may constitute a secondary source of several AMs; e.g., waste obtained during the enrichment process of colemanite contains lithium, rubidium, and cesium [22]. Cesium, like Li and Rb, can also be recovered from desalination brines [23].

Valuable metals can also be recovered from different types of waste (e.g., waste from ore processing, waste electrical and electronic equipment (WEEE), etc.). Such processes can be carried out using various methods (e.g., pyrometallurgical, hydrometallurgical, bio-metallurgical), but due to numerous advantages (e.g., satisfactory performance, scalability, relatively low energy consumption, low carbon dioxide emissions, etc.), hydrometallurgical processes, which rely on chemical reactions in various leaching media, are increasingly used for this purpose [24,25,26]. However, before the application of hydrometallurgical methods to some types of waste (e.g., WEEE), additional pre-treatment steps are necessary for maximizing metal recovery (e.g., dismantling and crushing the material, removing glass, etc.) [27]. Both in the case of solutions obtained as a result of leaching various types of waste or ores, as well as in the case of brines or wastewater containing metal ions (e.g., AMs), in order to recover valuable metals (or remove environmentally hazardous metals), it is necessary to use efficient and selective separation techniques. The separation of metal ions from different solutions is most often carried out using physical methods (e.g., membrane filtration, adsorption) or chemical methods (chemical precipitation, adsorption, ion exchange, cementation, etc.), or a combination of various techniques. The wide variety of recovery methods currently in use results from the fact that most of them are performing well only when retrieving specific metals from specific solutions, whose properties may differ significantly (depending on the type of waste/ore from which the solution was obtained by leaching, the type of leaching agents used, etc.). Moreover, when it comes to the developed recovery methods, economic aspects are also important (e.g., the recovery cost should not exceed the value of the recovered metals), as well as environmental consideration (e.g., the implemented solutions should be consistent with the principles of sustainable waste management, recovery technologies should not pose a threat to the environment, etc.) [25,26]. Since the use of hydrometallurgical techniques for the sourcing of metals often requires the use of leaching agents (acids or alkalis) and sometimes also complexing compounds, which can be hazardous for the environment, and because the processes are not always sufficiently efficient and selective, there is a growing demand for novel methods that offer better performance and are more environmentally friendly [20,21]. Environmentally safe solutions can involve various modifications of traditional methods, such as, for example, the use of more neutral, non-toxic solvents for leaching (e.g., deep eutectic solvents) or the introduction of the so-called fusion technologies that incorporate additional processes (e.g., photocatalysis), which are usually more complex [28,29]. One of the dynamically developing research areas in this field is the search for novel, effective, and safe materials that could be used in adsorption processes intended for separating less common AMs.

This paper presents an overview of the latest achievements (mainly from 2023 to 2024) in the field of recovery or removal of lithium, cesium, and rubidium ions from various solutions (e.g., seawater, brines, leachates obtained from ores and wastes) using novel adsorption materials. Particular attention was paid to the performance of the developed materials, the possibility of their implementation on a larger, industrial scale in the future, as well as their potential impact on sustainable development and environmental safety. The advantages and disadvantages of adsorbents materials designed for lithium ions recovery from leachates of used batteries were also discussed, focusing mainly on those developed in 2024, as earlier materials have been analyzed in other review articles [29].

## 2. Adsorption Methods

Generally, adsorption can be defined as a surface phenomenon in which specific substances are transferred from a liquid or gas phase to the surface of condensed phase, forming a superficial layer. Although adsorption can occur at different phase interfaces (e.g., liquid–solid, liquid–gas, solid–gas), in the case of separation of metal ions from various solutions, the processes taking place at the interface of the liquid and solid phases or liquid and liquid condensed phases are of key importance. The condensation and deposition of atoms or molecules of the adsorbed substance on the surface of the adsorbent can occur through weak physical interactions (e.g., Van der Waals interactions, London forces, dipole–dipole attraction), which is referred to as physical adsorption. Alternatively, when chemical reactions occur (e.g., the formation of covalent or ionic bonds), the process is known as chemical adsorption. Usually, physical adsorption is a non-specific reversible process (the reverse process being desorption), while chemical adsorption is a highly specific, often irreversible process. The mechanism of the adsorption process and its effectiveness depend on many factors, such as the properties of the adsorbent (e.g., surface porosity, presence of specific functional groups), its amount, the nature and concentration of the substance being separated, the composition of the solution (e.g., the presence of co-existing ions and their properties), the pH of the solution, the temperature, the time of the adsorption process, etc. [30,31]. The large number of factors influencing the adsorption processes and their mutual dependencies means that determining the optimal conditions for the separation process of specific metal ions from specific solutions is usually time-consuming and labor-intensive. However, adsorption processes are characterized by several attributes that give them a technical advantage over other processes used for the separation of various metal ions. In general, adsorption is a relatively inexpensive process; without special electrical requirements (unlike electrochemical techniques), it is not prone to the chemical resistance issues (often observed in membrane separation), offers greater selectivity, and can be integrated with the existing industrial processes [32]. The key issue related to adsorption performance in relation to metal ions is the appropriate selection of the adsorbent, which should not only enable the process to be carried out with satisfactory efficiency and selectivity but should also offer other advantages, such as non-toxicity, stability, and the possibility of multiple use after regeneration. These additional benefits of adsorbents may have significant implications for environmental protection and sustainable development, especially in relation to adsorption processes carried out on an industrial scale. Currently, various mineral, organic, and biological substances are used as adsorbents, including zeolites, clay minerals, activated carbons, agricultural wastes, and polymeric materials [33,34]. Depending on their nature, adsorbents can be categorized in various ways, the most common distinction being between organic and inorganic adsorbents. Organic adsorbents used for the separation of alkali metal ions include, for example, polymers containing active ion exchange groups (organic ion exchange resins), whereas inorganic adsorbents encompass ion sieves, aluminum salts, as well as modified and natural mineral adsorbents [35].

## 3. Ion Sieves

**Figure 1:**
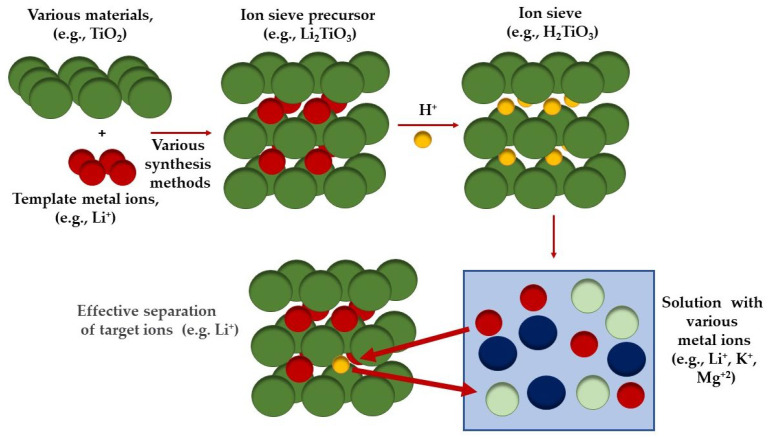
A simplified scheme of the preparation and operation of the ion sieve (prepared on the bases of [38,39]).

A specialized group of adsorbents are the so-called ions sieves (ISs), which are currently mainly used for the separation of lithium ions from various solutions (e.g., model solutions, sewage, brines, waste leachates). Lithium ion sieves (LISs) based on titanium or manganese, thanks to their unique microscopic lattice structure, are usually characterized by high efficiency, selectivity (also on a macroscopic scale), excellent stability in acidic environments, and the possibility of multiple use. Titanium-based LISs, usually classified into two groups, of sieves with spinel structure (H_4_Ti_5_O_12_) and with layered structure (H_2_TiO_3_), are synthesized using TiO_2_ or titanium-containing metal organic materials by using various reagents and synthesis methods (e.g., solid-state reactions, hydrothermal and sol–gel methods). Usually, the simplest process of generating lithium ion sieves is carried out in two steps: in the first step, a lithium ion sieve precursor is produced (e.g., Li_2_TiO_3_), then Li^+^ is eluted (e.g., using acids) to obtain the lithium adsorbent (e.g., H_2_TiO_3_) [36,37]. Figure 1 shows a simplified scheme of the preparation and operation of the ions sieve.

### 3.1. Lithium Ions Separation

The field related to the designing and synthesizing of new ion sieves intended for the recovery of Li^+^ from various solutions is developing rapidly, driven by the need to increase the adsorption capacity and selectivity of new materials while also shortening processing times and increasing the number of cycles in which the adsorbent can be used after regeneration. The adsorption efficiency of LISs can be influenced in various ways, e.g., by modifying the ion sieve synthesis methods, which can impact factors such as the crystal size, precursor aggregation, and the immobilization of the sieve material in different forms (e.g., beads, foams, membranes, nanofibers) in order to mitigate the structural dissolution of the sieves and improving their stability [37].

In recent years, several modifications have been made to the synthesis methods of ion sieves to improve their performance. For example, Li et al. [36] used amorphous TiO_2_ for the synthesis of titanium-based lithium ion sieve H_2_TiO_3_ (HTO) and reported that the generated HTO-Am enabled the recovery of 96.53 % of lithium ions from a single-component solution. Furthermore, they showed that the presence of coexisting ions in the solution (i.e., K^+^, Na^+^, Mg^2+^) had only a slight effect on the adsorption of lithium and that the adsorbent maintained consistent adsorption capacity after five consecutive cycles. Liu et al. [40] introduced a spray-drying step to the solid-state synthesis method in order to facilitate the transformation of the solution into solid powder and improve the uniformity of the generated precursors. This modification resulted in well-dispersed, spherical, layer-structured H_2_TiO_3_ ion sieve particles. They found that the prepared HTO used for lithium ions recovery from saline solutions exhibited a high adsorption capacity (30.08 mg/g) and an ultra-low titanium dissolution loss. Additionally, it was suitable for repeated use (with an adsorption capacity of about 26 mg/g over 5 cycles of adsorption/desorption processes). Sun et al. [41], who used surfactants (a double surfactant system consisting of the triblock copolymer PEO_106_-PPO_70_-PEO_106_ and hexadecylamine) during the synthesis of titanium-based ion sieves in order to control their morphology (surfactants easily form micelles in solution, which reduce the surface tension and affect the number and structure of pores in the produced material), reported that such HTOs are characterized by great hydrophilicity and enable efficient lithium ions recovery from liquid sources (in optimal experimental conditions, Li^+^ ions adsorption capacity was 56.03 mg/g). Moreover, the adsorbent maintained about 96% of its initial adsorption capacity after five consecutive cycles of adsorption/desorption.

Various materials can be used for shaping adsorbents powders, including different polymers (e.g., polyvinyl chloride, polyvinyl alcohol, polyacrylonitrile, polyvinyl butyral) or inorganic substances. For example, H_2_TiO_3_ powders shaped into granular form by polyvinyl butyral polymers with hydrophilic and hydrophobic properties (PVB-H_2_TiO_3_) have been successfully applied for efficient and selective recovery of lithium ions from various brine solutions with different pH levels and containing a range of co-existing ions [36]. Fangjie et al. [42] used attapulgite, an aluminosilicate clay mineral with a layered chain structure, as a binder and dispersant in the granulation process of titanium-based urea-doped lithium ion sieves generated by the modified high-temperature solid-phase method. They reported that the obtained material exhibited high efficiency (adsorption capacity of Li^+^ was about 48 mg/g) and selectivity towards lithium ions (in the presence of coexisting Na^+^, K^+^, Mg^2+^, and Ca^2+^ ions), was stable, and could be used five times without significant loss of its adsorption activity. Chen et al. [43] developed a granulated titanium-based ion sieve intended for lithium recovery from geothermal waters using agar as a spherality-shaping and sacrificial porogenic agent. The obtained material with a uniform mesoporous structure was efficient (the adsorption capacity towards Li^+^ ions was about 26 mg/g), highly selective (geothermal waters also contain Na^+^, K^+^, Mg^2+^, Ca^2+^ ions), and showed low dissolution loss. For producing mechanically stable composite LISs, various types of biomaterials (e.g., chitosan, cellulose, agar) can be used, since, due to their structure and properties, such substances enable the creation of three-dimensional bio-composite materials. The possibility of using LISs modified with biomaterials for the recovery of lithium ions from various solutions may offer economic advantages, for example, when carrying out processes on a larger scale (biomaterials are generally affordable and widely available) and may have a positive impact on the natural environment and on sustainable development (efficient lithium recovery, biomaterials are non-toxic, possibility of using recycled biomaterials) [44].

However, in the case of lithium ions recovery from solutions using LISs, the performance of the adsorbents is influenced by the process conditions. For example, Zhu et al. [45] showed that a self-made titanium-based lithium ion sieve used to recover lithium ions from West Taijinar brine achieved significantly higher adsorption after the adjustment of ammonia buffer (with the addition of ammonia, the adsorption capacity was about 19 mg/g at 30 °C for 24 h, representing an increase of about 87% compared to results obtained for experiments conducted without ammonia). They reported that the NH_3_·H_2_O-NH_4_Cl buffer system used increased the number of active adsorption sites and accelerated the H^+^/Li^+^ exchange rate. Recently, Tang et al. [46] examined the effect of NaHCO_3_ buffer addition on direct lithium ions extraction from Tibetan brine using HTO ion sieves. They found that the added buffer reduced the aggregation of H^+^ and stabilized the pH value (at around 7.5), which accelerated the mass transfer rate of lithium ions in the initial stage of the adsorption process. Extensive research on the use of HTO ISs continues to explore various aspects of the lithium ions adsorption processes. Examples of results published in 2024 regarding the application of various titanium-based ion sieves (and hydrogels loaded with HTO) for the separation of lithium ions from aqueous solutions presented in Table 1 reflect the latest developments in this field.

One of the recent research trends in enhancing LISs focuses on incorporating soft carbon materials, which are characterized by high specific surface area. For example, Qian et al. [47] used graphene oxide (GO), a soft carbon characterized by a two-dimensional lattice porous structure and high dispersion performance, to produce HTO@GO ion sieve via a two-step process (sol–gel co-mixture and a solid-state calcination). They reported that GO effectively reduced the agglomeration of LTO, which resulted in smaller particle sizes. Additionally, the introduction of GO also increased the content of isolated OH groups in HTO@GO, which led to increased Li^+^ ions adsorption capacity of the ion sieve (about 38 mg/g). Importantly, the efficiency of the adsorption process did not decrease significantly after using the adsorbent in six consecutive cycles (the adsorption capacity decreased to about 33 mg/g). Recently, Lin et al. [55] modified a lithium-ion sieve HTO by incorporating biomass carbon aerogel (BCA), derived from citrus peel), which helped to improve the surface hydrophilicity of the HTO@BCA adsorbent, increased specific surface area, and increased contact surface between the lithium ions solution and the adsorbent. This modification resulted in a highly effective adsorption material with a high lithium ion adsorption efficiency after 5 consecutive cycles (36.6 mg/g), as well as with high selectivity towards Li^+^ in a solution containing Na^+^, K^+^, Ca^2+^, and Mg^2+^ ions (the increased lattice spacing caused by BCA enhanced the adsorption selectivity for small-sized ions). In terms of sustainability, this method offers two advantages: the possibility of efficient recovery of valuable lithium, as well as utilization of biomass carbon from organic waste.

Another effective approach to improving the adsorption capacity of HTO-based ions sieves by regulating their morphology is doping with various elements, such as rare earth elements (REEs), which can effectively modify interface properties. For example, Liu et al. [51] synthesized Nd-doped HTO (Nd-HTO-1%) and found that Nd-doping promoted the formation of oxygen vacancies and increased the density of surface hydroxyl groups, resulting in a highly hydrophilic adsorbent surface. Additionally, Nd-HTO-1% exhibited high selectivity for lithium ions, was characterized by high adsorption capacity even after eight consecutive cycles (39.69 mg/g), and was also stable (with minimal titanium loss observed during testing). Highly selective and efficient lithium ions sieves have also been obtained by doping HTO with elements, such as lanthanum, copper, cobalt, tungsten, or magnetic Fe_3_O_4_ nanoparticles [50,56,57,58,59].

Recently, there has also been a trend towards creating of more complex, composite ion sieves in order to improve their performance. For example, studies have shown that the use of a hybrid binder (cellulose acetate/sulfonated poly(ether ketone)/poly(vinyl chloride)) in the synthesis of granulated lithium ion sieves increases the adsorption capacity and the regeneration capability of the granulated HTO-LIS [53]. Lin et al. [60] synthesized lithium-titanium-zirconium composite oxide via a solvothermal method, converting it into a lithium-ion sieve, H_4_Ti_4.98_Zr_0.02_O_12_ (HZrTO). They reported that this novel material can be successfully used in lithium ions recovery from a mixed solution of Li^+^, Na^+^, K^+^, Mg^2+^, and Ca^2+^ (the exchange capacity of HZrTO for Li^+^ was up to 6.99 mmol/g and remained at 6.76 mmol/g after five exchange cycles in a mixed solution of 6.23 mmol/g). A specific example of a complex adsorbent with titanium-based LIS is the hydrogel loaded with HTO lithium-ion sieve. Zhang et al. [54] developed a novel method for hydrogel synthesis through free radical polymerization, using titanium-based lithium-ion sieves, acrylamide, and polyvinyl alcohol, and reported that the resulting HTO-PVA/PAAm adsorbent exhibited a soft three-dimensional network, adjustable pore structure, high specific surface area, and suitable hydrophilicity. The application of HTO-PVA/PAAm hydrogel enabled efficient adsorption of lithium ions from aqueous LiCl solutions (the efficiency of the process depended on the pH, with adsorption capacities of about 22 and 31 mg/g in solutions of pH 7.2 and 12, respectively), as well as efficient desorption (90% of adsorbed Li^+^ was desorbed within 1 h under optimal experimental conditions). Moreover, the hydrogel maintained the Li^+^ adsorption capacity at the level of 90% after eight adsorption–desorption cycles, with minimal titanium loss (<0.42% per cycle). It can be assumed that there will be an increasing interest in adsorbents of this type, because, as it has been shown, they are characterized not only by excellent lithium ion adsorption capacity, relatively short process time, and easy and efficient desorption, but are also non-toxic and environmentally friendly, so they can potentially be used on a wider scale in the future, e.g., for the adsorption of lithium ions from brines of salty lakes and sea water.

For manganese-based ions sieves, typically generated from spinel lithium manganese oxide (LMO) precursors for adsorbents (e.g., LiMn_2_O_4_, Li_4_Mn_5_O_12_), the main challenges to their applications are associated with difficulties in their control during adsorption processes and recovery. Hence, ongoing research focuses on the possibility of modifying ion sieves in such a way as to enhance their stability and durability and facilitate their separation from the solution while at the same time maintaining high efficiency. For example, it has been reported that Fe_3_O_4_-doped magnetic lithium ion-sieve LMO/FO enabled not only more efficient separation of lithium ions from brine solution containing Li^+^, Na^+^, K^+^, Mg^2+^, and Ca^2+^ (adsorption capacity of Li^+^ ions was 29.33 mg/g) compared to undoped LMO LIS but was also more chemically stable [61]. Magnetic ion sieves are relatively easy to recycle, but their performance depends on many factors. Gao et al. [62], who synthesized magnetically recyclable Fe-doped manganese oxide lithium ion sieves with a spinel-structure using a solid-state reaction method, showed that the adsorption capacity of such LISs towards lithium ions depends on factors such as calcination temperature, calcination time, Fe doping amount, solution pH value, initial Li^+^ concentration, and adsorption process temperature. Under optimal experimental conditions, the adsorption capacity of the Fe-doped LIS reached 34.8 mg/g. Particularly important, especially in the context of the potential application of this type of ISs on a larger, industrial scale in the future, is the possibility of recovery of lithium ion-sieve through magnetic separation in a magnetic field. Moreover, after regeneration, such LIS can be successfully used several times (although the efficiency drops to 70% after 5 consecutive adsorption/desorption cycles). However, ongoing research focuses on improving the performance of magnetic LISs, and different substances are used as doping agents. Han et al. [63] synthesized magnetically recyclable Al-Fe co-doped and solely Al-doped manganese oxide LISs, reporting that the obtained Al-Fe co-doped HMO reached an adsorption capacity of about 45 mg/g, whereas the Al-doped material was about 33 mg/g. Moreover, the adsorption capacity of Al-Fe-doped LIS remained above 95% after five cycles. Given that doping is one of the most effective ways to regulate the composition and lattice structure of manganese-based lithium ion sieves (by stabilizing the spinel structure of LMO), intensive research is being carried out on the possibilities of using various substances for this purpose, including B, Al, Mg, Cr, Ga, and Co. The main goals of LISs modifications by doping are to increase the efficiency of lithium ions adsorption, to inhibit the Mn dissolution, and to improve the cyclic stability of the adsorption material [64,65,66,67,68].

### 3.2. Cesium Ions Separation

Although ion sieves of both types (titanium- or manganese-based) have primarily been used in lithium ions adsorption processes aimed at recovering valuable Li, in recent years attempts have also been made to utilize such types of adsorbents to separate radioactive cesium ions, considered to be one of the most dangerous environmental pollutants, from aqueous solutions. The threat to the environment in the case of Cs is related to the fact that some cesium isotopes (^134^Cs and ^137^Cs) are not only hazardous (radioactive) to humans and animals, but they are also highly soluble and volatile and are usually present in nuclear wastewater in considerable quantities. Therefore, it is necessary to remove these ions from wastewater to prevent their widespread dispersion in the environment [69]. Recently developed cesium ions sieves differ from traditional titanium-based ISs, and the introduced modifications concern, among others, the addition of substances not previously used for this purpose or/and changes in the synthesis processes, both aimed at improving the properties of the adsorbent and its performance. For example, Geng et al. [70] synthesized Cs_2_Ti_6_O_13_ using sol–gel and de-templating methods, with polymethyl methacrylate (PMMA) polymer microspheres as templates. Using such precursors with diluted HCl elution produced porous, nanosheet cesium ion sieves (HTO/PMMA). The sol–gel method is particularly effective due to its straightforward reactions and the ability to achieve high crystallinity in the final product. The important advantages of PMMA are easy processing and modification, precise size control, and the ability to thermally decompose into small molecule gases (e.g., CO_2_, H_2_O), making it an effective removable template. The obtained results have shown that HTO/PMMA cesium ISs, in comparison with HTO prepared by the direct sol–gel method, exhibited a larger specific surface area and a higher number of exchange sites for cesium ions, higher adsorption capacity (about 224 mg/g for HTO and about 299 mg/g for HTO/PMMA), and satisfactory recyclability (with no significant decrease in adsorption capacity after 5 cycles of adsorption/desorption). Moreover, it has been reported that the adsorption process involving the cleavage of O–H bonds and the formation of O–Cs bonds (ion exchange reactions) was spontaneous and exothermic. The process was also more effective at lower temperatures, which was conductive to adsorption in the H^+^- Cs^+^ exchange system. Due to the properties of this novel type of ion sieves, HTO/PMMA may be an efficient material for recovering radioactive Cs^+^ ions. Recently, Yang et al. [69] synthesized a series of cesium ion sieves precursors, Cs_2_Ti_6_O_13,_ using a high-temperature solid-phase method (CTO-1), a solvothermal method (CTO-2), and a soft template-assisted solvent thermal method (CTO-3, composed of loose nanosheets), using cesium carbonate as the cesium source and tetrabutyl titanate as the titanium source. Additionally, in the template method, they used the triblock copolymer PEO_106_-PPO_70_-PEO_106_ (F127, Mw = 12600) and hexadecylamine as template agents to modulate the surface morphology (increasing the specific surface area) and hydrophilicity of the ion sieve precursor. They reported that the cesium ion sieve (H_2_Ti_6_O_13_, HTO-3) obtained via the template method, after hydrochloric acid treatment, exhibited a large specific surface area and good hydrophilicity. Due to the structure favoring ion exchange between Cs^+^ and H^+^, the adsorption of cesium ions by HTO-3 was fast and highly efficient (in 2h the adsorption capacity was about 360 mg/g). Moreover, this novel adsorbent performed better than the corresponding materials obtained by the other synthesis methods (HTO-1, HTO-2), exhibited good selectivity towards cesium ions (in the presence of Li^+^, Na^+^, K^+^, and Rb^+^ ions in the solution), and maintained stability (the adsorption capacity decreased by only about 13% after five regeneration cycles). Additionally, it has been shown that the adsorption process efficiency depended on experimental conditions (pH, solution concentration, temperature, etc.). The adsorption mechanism of cesium ions was identified as monolayer and chemical adsorption. Based on the obtained results, the authors concluded that HTO-3 shows significant potential for industrial application in the recovery of cesium resources.

## 4. Aluminum-Based Adsorbents

### 4.1. Lithium Ions Separation

**Figure 2:**
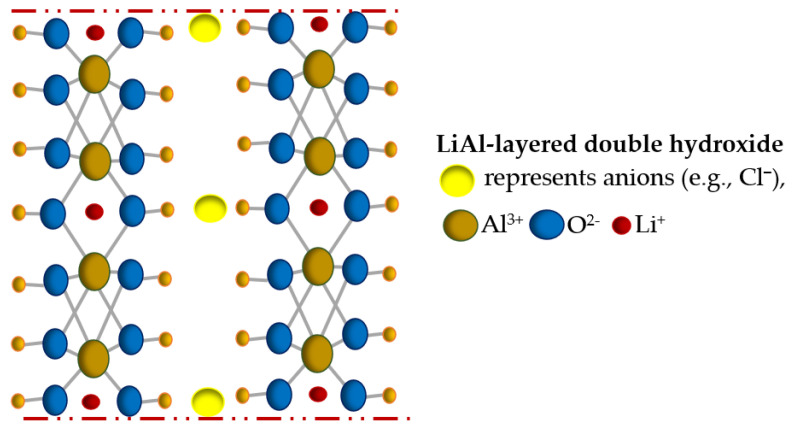
A simplified scheme of the structure of lithium-aluminum layered double hydroxide (prepared on the basis of [73]).

Aluminum-based adsorbents are currently widely used for the recovery of lithium from various types of brines. Of particular interest are lithium-aluminum layered double hydroxides (Li/Al-LDHs), used in direct lithium extraction (DLE) technology due to their low environmental impact. Li/Al-LDH type adsorbents are characterized by a disordered layered structure with the general chemical composition of LiX mAl(OH)_3_ nH_2_O, where X represents the anions (e.g., Cl^−^), m corresponds to the number of aluminum hydroxide molecules (e.g., 2), and n denotes the number of water molecules. The structure of Li/Al-LDHs (presented in Figure 2) is stabilized by strong covalent bonding forces between the adsorbents layers, as well as electrostatic forces, van der Waals forces, and hydrogen bonds between the layers. Lithium ions adsorption is related to the ability to occupy sites in the octahedral cavities of Al(OH)_3_ (with diameters similar to those of Li^+^), while the anions remain in the interlayer space (electrical neutralization). Eluting of lithium ions with a neutral solution results in the formation of an efficient adsorbent characterized by high selectivity towards lithium ions (due to steric hindrance, larger ions (e.g., Na^+^, K^+^, etc.) cannot be adsorbed) [71,72].

In addition to high efficiency (usually >90%) and selectivity, the advantages of this type of adsorbents include relatively simple synthesis methods (e.g., solid-state synthesis, hydrothermal method), no need to use hazardous chemical compounds (use of LiCl, LiOH, or Li_2_SO_4_ as a source of lithium ions and naturally occurring minerals, e.g., gibbsite (sources of aluminum hydroxide)), and the possibility of repeated use of the adsorbent after lithium ions elution (e.g., with water). In comparison with HTO and HMO-based adsorbents, Li/Al-LDH adsorbents are characterized by significantly greater durability (under optimal conditions, they can be used for several hundred consecutive adsorption–desorption cycles) and relatively low production costs. In addition, they have been successfully implemented for commercial applications [71]. However, the performance of Li/Al-LDH adsorbents in lithium ions recovery depends on both the structure and properties of the separation materials (e.g., adsorbent structural stability, type of interlayer anions, which affects the de-embedding rate of lithium ions) as well as on the conditions of the adsorption processes (e.g., composition and properties of brine, desorption temperature, desorption solution dosage, pH, etc.). Since these types of adsorbents may perform differently in various environments, ongoing research aims to improve the adsorption properties of aluminum-based adsorbents under different experimental conditions (e.g., in sulfate-type salt lakes where the recovery of lithium ions may not be satisfactory due to the presence of interlayer SO_4_^2−^ anions, which hinder effective desorption) [72]. Table 2 presents examples of novel aluminum-based adsorbents developed over the last two years for the separation of lithium ions. These examples reflect the current research trends in the modification of these types of adsorbents.

Recent studies on increasing the adsorption capacity of aluminum-based LDHs adsorbents have focused, among other factors, on the effect of anions intercalation or cations doping. It has been shown, for example, that doping with various cations (e.g., Fe^3+^) can reduce the Li^+^ diffusion energy barrier in adsorbents and enhance the material stability [81]. However, the modifications of this type of adsorbent can be more complex, involving both the interlayer anions and cation doping. For example, Huo et al. [76] developed a one-pot synergistic modification method based on ion-doping technology to synthesize a novel Co-LDHs-SO_4_ adsorbent characterized by excellent adsorption capacity and cycling stability in various lithium ions solutions. They reported that the advantages of this adsorbent stem from the two contributions of anions, which enhance affinity with the positively charged [LiAl_2_(OH)_6_]^+^ layer, and cations, which improve the stability of the Al-O skeleton. Importantly, they also demonstrated that this type of adsorbent enables the extraction of Li^+^ from sulfate-type salt lakes, which is not a typical feature of such materials (usually in the case of LDHs-type adsorbents with interlayer Cl, the ion exchange reaction between SO_4_^2−^ ions from the solution and Cl^−^ in the adsorbent causes structural distortion). Understanding the mechanisms behind the poorer performance of aluminum-based lithium adsorbents in sulphate or carbonate brines is essential for developing solutions to improve recovery efficiency. Such studies often involve advanced methods, including quantum chemistry (e.g., DFT methods) [82,83].

Modifying adsorbents to enable their effective operation in various environments could significantly expand the application of aluminum-based adsorbents in the future. However, current research is focused not only on understanding the behavior of aluminum-based adsorbents in various conditions but also on the influence of the applied synthesis method on the performance of such materials. For example, Li et al. [77] showed that in the case of aluminum-based adsorbents prepared by the precipitation method, their stability and efficiency are influenced by factors such as pH modulation at the end stage and the maximum desorption capacity of Li^+^ during the elution process. Some of the recent studies have also focused on the synthesis of granulated adsorbents [74,75,78], as their form has practical importance, e.g., in industrial applications (easier separation and recovery than powdered aluminum-based adsorbents). However, since the granulation process is usually quite complicated, involves higher costs, and sometimes adversely affects the adsorption efficiency, attempts have also been made to dope the LDHs with superparamagnetic nanoparticles (Fe_3_O_4_@SiO_2_) to achieve rapid recovery of adsorbents using a magnetic field [80]. A key advantage of magnetic aluminum-based LDHs adsorbents, apart from their efficiency, is that they can be efficiently recovered (with over 90% efficiency) within a few to several minutes, leveraging their superparamagnetism [84].

### 4.2. Cesium Ions Separation

Despite a number of advantages (e.g., simple preparation, stability, outstanding intercalation characteristics, etc.) and wide applicability in lithium ions separations, LDHs adsorbents generally lack a strong affinity for cesium ions (which is due to the structure of such adsorbents and the properties of the cesium ions). However, attempts have been made to modify this type of adsorbents in such a way as to enable efficient and selective removal of hazardous cesium ions from aqueous solutions. A possible solution is to modify LDHs with Prussian blue (Fe_4_[Fe(CN)_6_]) analogues (PBAs), which are known for their decent adsorption selectivity and affinity toward Cs^+^. However, due to a number of problems related to their conventional use in cesium ions adsorption (e.g., poor water stability, problems with separation and recycling of ultrafine PBA powder, insufficient performance in fixed-bed adsorption experiments), these compounds are often grafted or immobilized in substrates with different functionalities (e.g., various LDHs) [85]. For example, a Prussian blue analog was used to modify copper–aluminum layered double hydroxide, and the novel adsorbent (PBA@CuAl-LDH) synthesized via a one-step method was successfully used for the removal of radioactive Cs^+^ from wastewater (adsorption capacity of 109.2 mg/g, 85% of PBA@CuAl-LDH was recycled) [86]. Recently, it has been shown that intercalation of potassium ferrocyanide (HCF, K_4_Fe(CN)_6_) into layered double hydroxide Mg/Al-LDH through the co-precipitation method leads to the formation of HCF@LDH adsorption material. This material has been used both for separating Cs^+^ ions from model solutions and seawater, enabling efficient and selective removal of cesium ions (100% and 90%, respectively). Moreover, the recycled adsorbent maintained its adsorption efficiency over five consecutive cycles. The analysis of the adsorption mechanism showed that cation exchange between K^+^ and Cs^+^ played an important role in effective cesium ions adsorption, with the interstitial spaces of the hexacyanoferrate crystals facilitating the cation exchange process [87].

## 5. Mineral-Based Adsorbents

**Figure 3:**
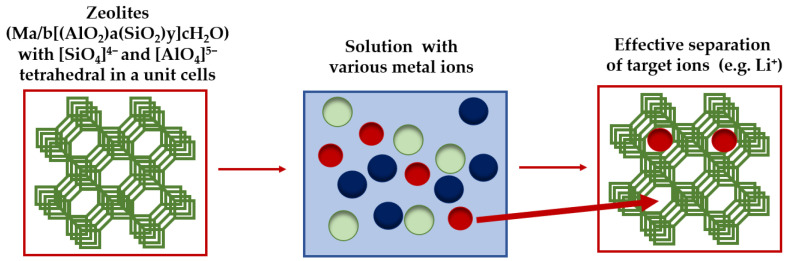
A simplified scheme of the separation of metal ions by zeolite adsorbent (prepared on the bases of [90]).

Adsorbents based on various types of minerals are of great interest due to a number of advantages, including the ability to separate metal ions, easy accessibility of raw materials, relatively low production costs of adsorbents, and their high stability. Metal ions separation methods based on such adsorbents are usually efficient, environmentally safe (green), and relatively easy to carry out. One such group of minerals used to produce such types of adsorbents intended for the recovery or removal of various metal ions (including Li^+^, Cs^+^) from solutions are zeolites (crystalline aluminosilicate materials), which are known for their high ion selectivity and exchange capacity [88,89]. In general, both natural and synthetic zeolites exhibit nano porous properties and are composed mainly of silica tetrahedral units linked with alumina tetrahedral units (with general composition Ma/b[(AlO_2_)a(SiO_2_)y]. cH_2_O, where M—an alkali or alkaline earth metal cation, b—the valence of earth metal cation, c—the amount of water molecules per unit cell, a and y—the total number of the [SiO_4_]^4−^ and [AlO_4_]^5−^ tetrahedral in a unit cell of the zeolite) [90]. The open crystal structures of zeolites allow for chemical substitution between silicon and aluminum ions within their lattice positions, creating a regular negatively charged surface. Currently, in addition to zeolites obtained from natural deposits, synthetic zeolites in various forms (e.g., zeolites-A, zeolites-P, zeolites-X, and others), which are characterized by high purity, large surface area, and ordered porous structure, are used in the adsorption processes of various pollutants from aqueous solutions. However, their synthesis often involves complex steps [91]. Interestingly, zeolites can also be synthesized using aluminosilicate residues obtained from lithium production processing of ores [92,93], helping to reduce the amount of generated waste. In the past two years, various zeolites, including commercially available ones, have been successfully and efficiently used for the recovery of valuable lithium or the removal of radioactive cesium ions from different aqueous solutions [88,94,95,96]. Figure 3 shows a simplified scheme of the separation of metal ions by zeolite adsorbent. It has been shown that adsorption materials of this type, due to their high ion exchange capacity and excellent stability (including hydrothermal, mechanical, and radiation stability), can be used for the treatment of nuclear wastewater (which may contain, in addition to radioactive cesium, also strontium, cobalt, uranium, plutonium, americium, samarium, or europium ions) [97].

Despite the numerous advantages of zeolite adsorbents, ongoing research aims to further improve their performance. For example, there are studies exploring the effects of different modifications in the synthesis process (e.g., the use of cold sintering) [95], the material form (e.g., the influence of the size of the aluminosilicate grains on cesium ions adsorption efficiency) [98], and methods to facilitate the separation and reuse of the adsorbent (e.g., the formation of magnetized zeolites) [99]. So far, zeolites have not been widely used for the separation of rubidium ions, but, due to the similar ionic radius of Cs and Rb, it can be expected that zeolites would also be effective in the capture of Rb [100].

It should be noted, however, that various minerals are currently being explored for the separation of lithium and cesium ions from aqueous solutions. For example, natural clay minerals like bentonite, attapulgite, and kaolinite have been used as eco-friendly adsorbents for removing Cs-137 from actual radioactive wastewater [101,102]. The possibility of effective removal of radioactive cesium from wastewater using clay adsorbents can be attributed to their specific properties. For instance, attapulgite is characterized by small particle size, a high specific surface area, good cation exchangeability, and effective functional sites. Attapulgite-based adsorbents not only proved to be effective in removing Cs-137 (adsorption efficiency > 95% in a 2h process), but they are also inexpensive and easily available [103]. However, there is also research aimed at increasing the adsorption capacity of raw clay materials through activation. One of the activation techniques used is inorganic acid treatment, which, in general, results in material with a larger specific surface area, better porosity, and more active sites compared to raw clay. Recently, studies have shown that acid-activated clay can be successfully used for the removal of radioactive Cs^+^, Sr^2+^, and Co^2+^ from water systems [104]. In response to the growing interest in adsorbents based on natural raw materials, complex adsorbents, so-called composites, which may contain several different components (e.g., natural clay montmorillonite, zeolite, chitosan, cross-linking agents, and plasticizers), are increasingly being developed to remove various metal ions, including cesium ion [105]. Research is also being conducted on the potential use of not only the minerals themselves but also specific mineral-type chemical compounds in the adsorption process. For example, the dittmarite (phosphate mineral) type magnesium phosphates (KMgPO_4_·H_2_O, (KMP)) and (NH_4_MgPO_4_·H_2_O, (NMP)) have been synthesized and applied for the adsorption of cesium ions from aqueous solutions. It has been shown that both adsorbents, KMP and NMP, exhibited remarkable adsorption capacities for cesium ions (630 mg/g and 711 mg/g, respectively), which were the highest among all reported adsorbents. After the adsorption processes, both adsorbents were structurally transformed into struvite-type CsMgPO_4_·6H_2_O, with two possible stacking structures (cubic or hexagonal), which depend on the solution pH [106]. KMP has also been utilized in fiber-supported layered magnesium phosphate adsorbents, which exhibited high cesium uptake capacity (about 299 mg/g) and Cs ions removal efficiency (93.8%) and therefore can be potentially useful for the decontamination of radiocaesium-contaminated waters [107]. Recently, magnesium ammonium phosphate adsorbent has been synthesized and applied for the removal of rubidium and cesium ions from simulated brine solutions. The obtained results demonstrated high adsorption capacities of the adsorbent (2.83 mol/g for Rb^+^ and 4.37 mol/g for Cs^+^) and its excellent selectivity towards cesium ions (primarily through an ion exchange mechanism) [108]. Additionally, a microporous zeolitic-like sulfide material used for cesium ion removal showed not only high capacity (about 249 mg/g) but also good selectivity for Cs^+^ in competitive multiple-component solutions. It has also been demonstrated to be regenerable using a low-cost and eco-friendly method [109].

## 6. Complex and Composite Adsorbents

One group of inorganic ion exchange adsorbents is metal sulfide ion exchangers (MSIEs), usually characterized by high adsorption capacity and excellent stability, composed in their simplest form of cationic skeletons and S^2−^ ligands. In general, they can exist as layered structures, three-dimensional crystals, or porous amorphous materials, with their structures depending on the components used and the synthesis method applied [110]. Many attempts have been made to modify these adsorbents in order to increase their performance in specific conditions. As a result, various novel adsorbents based on metal sulfides have been synthesized (e.g., porous fiber-supported metal tin sulfide PVC-[Me_2_NH_2_](2)Sn_3_S_7_ with PVC as a support; cation-intercalated (by NH_4_^+^, Na^+^) lamellar MoS_2_; metal sulfide, Cs_2.33_Ga_2.33_Sn_1_·_67S_8·H_2_O with an “imprinting effect” on Cs^+^) and successfully used to remove cesium ions from aqueous solutions, such as simulated wastewater or brines [111,112,113]. Recently, a novel layered tungsten-doped metal sulfide, K_1.46_W_0.02_Sn_2.95_S_6.69_ (KWTS), was formulated and applied for the efficient and fast recovery of rubidium ions from wastewater. KWTS demonstrated a high adsorption capacity of Rb^+^ (184.68 mg/g, adsorption rate of 10 s) and selectivity towards rubidium ions over other AMs. Additionally, magnetizing and cross-linking KWTS with sodium alginate enabled the production of micron-sized spherical particulates of Fe_3_O_4_@KWTS, characterized by very good adsorption performance, easy separation after the adsorption process, and extraordinary stability after repeated cycles [110]. All these features of adsorptive materials are important; therefore, other adsorbents are also modified to improve these parameters.

Another group of substances used to produce adsorption materials for the separation of cesium and rubidium ions is metal ferrocyanides (MFCs), with an octahedral centered cubic structure. Ferrocyanides can be used as ions exchangers for the removal of cesium and rubidium ions due to their affinity for metal ions (Cs^+^ > Rb^+^ > NH_4_^+^ > K^+^ > Na^+^ > Li^+^). It has been shown that adsorbents of this type (i.e., K_2.01_[Mg_0.97_Fe(CN)_6_]·xH_2_O) enable efficient recovery of rubidium and cesium (90.7% and 97.9%, respectively) from ultra-high salt solutions [114]. The high efficiency of such adsorbents in separation processes carried out in concentrated, multi-component solutions is particularly significant for potential industrial applications, as wastewater, including radioactive wastewater, contains a number of different pollutants. An important issue in such applications is the possibility of easy separation and regeneration of the adsorbent from the solution after the adsorption process. Research is underway to develop suitable materials to address this challenge. For example, it has been reported that more complex, magnetic adsorbent MKNiCuFC@ZIF-8 (potassium nickel copper hexacyanoferrate generated on the surface of Fe_3_O_4_ coated with polydopamine (PDA) using an in situ approach and modified with zeolitic imidazolate ZIF-8) enabled not only efficient adsorption of rubidium ions (1030.69 mg/g at 45 °C) but also demonstrated excellent selectivity in multi-component solutions. Additionally, it could be easily separated using a magnetic field and reused multiple times after regeneration in a 1M KCl solution [115]. Recently, potassium cobalt iron cyanide double-hollow nanobubble prisms (Co-PBA DHNP) with controllable morphologies have been synthesized by using ZIF-67 hollow prisms as templates and Co-PBA double-shelled hollow prisms (Co-PBA DSHP). They were used for cesium ions removal from native brines. The results showed that the Co-PBA DHNP adsorbent exhibited a high adsorption capacity of Cs^+^ ions (494.38 mg/g), as well as high selectivity even in natural lake brine and excellent recyclability [116].

In general, various MOFs are highly suitable as supports for adsorbents intended for the recovery of lithium, cesium, and rubidium ions from aqueous solutions due to their diverse pore sizes (from angstrom scale to the nanoscale), high surface areas, regular pores, and good stability. Novel adsorbent composites are often based on metal-organic frameworks [117,118]. Adsorbents of this type can be synthesized/modified in various ways, and the implemented synthesis method can improve the properties of the adsorbent. For example, recently it has been reported that through the step-by-step introduction of building blocks into the MOF-based adsorbent, a gradual increase in material selectivity can be achieved. It has been shown that the highly selective and thermally regenerable adsorbent pNCE-SS@UiO-66 obtained in such a way (composed of 12-crown-4 (12CE4), sodium p-styrene sulfonate (SS), and the temperature-responsive trigger N-isopropyl acrylamide, encapsulated in the ion-sieving framework of UiO-66) applied for lithium ions recovery from synthetic brines enabled efficient adsorption of Li^+^, while leaving K^+^, Na^+^, and Mg^2+^ in the aqueous solution. What is important, after regeneration in water (at 40 °C), the adsorbent remained efficient and selective, making it reusable [117]. Lithium ions were also removed from aqueous solutions using an aluminum hydroxide isophthalate (i.e., CAU-10-H) MOF, which was synthesized via a solvothermal method using aluminum sulfate octahydrate and isophthalic acid as raw materials. It has been reported that the composite adsorbent demonstrated good adsorption and desorption performance under optimal conditions with an adsorption capacity of about 1.7 times higher than that of pure CAU-10-H. In general, MOF-based adsorbents offer several advantages, such as excellent hydrothermal stability, a large specific surface area, and easier regeneration, compared to conventional adsorbents [118].

In recent years, different MOFs-based materials have also been used to remove cesium and rubidium ions from various aqueous solutions. In particular, there has been growing interest in adsorbents capable of removing radioactive ^137^Cs and ^90^Sr ions, which are the most dangerous types of radioactive contaminants introduced into sea water (from nuclear waste and wastewater). MOFs adsorbents have been shown to be effective for this purpose. For example, it has been reported that mesoporous spherical Zn(4-hzba) MOF synthesized via the solvothermal method with 4-hydrazinyl benzoic acid (4-hzba) as a linker was used for the selective removal of Sr^2+^ and Cs^+^ from simulated seawater solutions, enabling fast and efficient adsorption (adsorption capacity of 275.2 mg/g for Sr^2+^ and 335.05 mg/g for Cs^+^, adsorption efficiency of 92.3% and 96.7%, respectively). In addition, the material demonstrated acceptable reusability (80% recovery of both ions after seven cycles). To better understand the adsorption mechanisms (chemisorption was the dominant process), quantum chemical methods were used (DFT method using Gaussian 16). Understanding the mechanisms of the reactions taking place during adsorption is important, as it facilitates the optimization of process conditions [119]. A zirconium-based metal-organic framework ([Zr_6_(µ_3_-O)_4_(µ_3_-OH)_4_(OBA)_4_(OH)_3_(H_2_O)_3_(Me_2_NH_2_)]_n_ (OBA = 4,4-oxy bis(benzoic acid); Zr(OBA) MOF)] has also been used for the removal of Sr^2+^ and Cs^+^ from solution. The adsorbent Zr(OBA) MOF exhibited high efficiency (adsorption capacities of 353 mg/g for Sr^2+^ and 432.91 mg/g for Cs^+^), enabling rapid adsorption of strontium and cesium ions. Moreover, it showed good reusability, making it a promising candidate for potential applications on a wider scale in the future, e.g., for the decontamination of Sr^2+^ and Cs^+^ from nuclear waste and seawater [120].

Research is also being conducted to increase the efficiency of MOFs-based adsorbents for the removal of cesium ions. Recently, radioactive cesium ions have been removed from nuclear wastewater using novel adsorbent ZIF-67 grafted into two-dimensional layers of transition metal carbides/nitrides (MXenes) Ti_3_C_2_. Utilization of this laminated adsorbent, which had increased the interlayer spacing and specific surface area of Ti_3_C_2_, with MXenes providing the main adsorption sites, enabled the removal of 96.2% of Cs^+^ within 6 h and was suitable for multiple use after regeneration. Moreover, due to structural advantages, the performance of the composite was significantly better than that of pristine ZIF-67 and Ti_3_C_2_, which are characterized by high specific surface area, good hydrophilicity, and abundant adsorptive sites [121].

One promising research area is the use of substances known for their selective adsorption properties towards specific metal ions for the production of composite materials, which can result in improved performance and additional advantages. For example, the advantages of the novel composite material (KCoFC@ZIF), which combines a metal-organic framework with potassium cobalt hexacyanoferrate (known for its high adsorption selectivity towards Rb^+^ ions, impaired in solutions containing high concentrations of K^+^), synthesized using a step-by-step method intended for rubidium ions removal from seawater, were not only a larger surface area (63% larger compared to KCoFC) and high Rb^+^ ions uptake with fast kinetics (eight-times higher compared to KCoFC) but also high efficiency in solutions containing elevated concentrations of potassium ions [122]. Due to their properties, MOFs can be used to produce various complex adsorbents that enable efficient and fast separation of lithium, cesium, and rubidium ions, as well as offer additional valuable advantages, such as the possibility of their use in various multicomponent solutions at different pH ranges, high stability, the possibility of multiple use after regeneration without significant deterioration of efficiency, etc. It is likely that in the future they will be used on a wider scale, including for the purification of radioactive wastewater and brines. Table 3 presents selected examples of complex and composite adsorbents developed over the last two years for the separation of AMs, reflecting current research trends in this field.

## 7. Ion Exchange Resins

Ion exchange resins (IERs), which operate through the Donnan membrane effect where a fixed charge attracts ions of the opposite charge into the resin pores, are of great interest in the context of the removal/recovery of various metal ions from aqueous solutions. Although they usually have limited selectivity and capacity for specific ions due to competition from other ions, which limits their adsorption efficiency, they can be relatively easily modified by introducing various substances into the polymer matrix [123]. Impregnated resins are composite materials consisting of polymer matrices and selective extraction agents that enable the binding of metal ions. They combine the advantages of both solvent extraction and ion exchange processes [124]. The growing interest in lithium, cesium, and rubidium ions removal methods has driven the development of resins designed for the efficient separation of these ions from specific solutions. Recently, various resins have been used for the removal of lithium ions from low-concentration Li^+^ solutions. For example, a resin functionalized with 2-(hydroxymethyl)-12-crown-4 ether has been synthesized and successfully used for selective adsorption of lithium ions from desilication solution of coal-based solid waste [125]. Additionally, a dibenzoylmethane/trioctylphosphine oxide-impregnated resin enabled the recovery of lithium ions from alkaline solution (a coal-based solid waste solution containing Li^+^, Na^+^, K^+^, AlO_2_^−^, SiO_3_^2−^). It should be emphasized that the synthesis of adsorbents with high Li^+^ selectivity and strong alkali resistance is important for the recovery of this valuable element from the pre-desilication solutions. Additionally, DFT calculations were also performed to investigate the lithium selective separation mechanism of β-diketone from the alkaline solutions [124].

Recently, ion exchange resins have also been used for the separation of rubidium and cesium ions from aqueous solutions. For example, Lee et al. [126] used Dowex G26 resin for the separation of Rb^+^ ions from the desalination brine (from the desalination plant in Taiwan). They reported that the Dowex G26-based separation process has advantages, such as high desorption efficiency and easy for scale operation, and disadvantages, such as a relatively low adsorption efficiency (86%) and enrichment ratio. In addition, the separation process of rubidium ions from the same brine was carried out using ionic liquid t-BAMBP/C2mimNTf2 (C2mimNTf_2_(1-ethyl-3-methylimidazolium bis(trifluoromethylsulfonyl)imide)), resulting in higher extraction efficiency (93%) and enrichment ratio (comparing to Dowex G26-based separation), but the process was more expensive and associated with continuous operation problems. Kim et al. [127] showed that ammonium molybdophosphate-polyacrylonitrile (AMP-PAN) resin (with custom-made sample-loading equipment) can be successfully used to concentrate radio-cesium from seawater. The advantages of the developed method, including reduced analytical time and increased sample throughput, make it a promising approach for the routine monitoring of radio-cesium in seawater. The AMP-PAN resin was also examined for its potential use in separating cesium ions from soil samples [128]. Ion-exchange resins have also been used to produce more complex and better-performing adsorbents. For example, polystyrene resins (PS) and zirconium phosphate (ZrP, a promising inorganic sorbent for radioactive cesium extraction) have been used to produce novel adsorbent ZrP-PS through facile confined crystallization in the host macropores. The ZrP-PS adsorbent exhibited remarkable Cs^+^ ions sequestration efficiency in both batch and continuous experiments (adsorption capacity 269.58 mg/g) and rapid equilibrium (80 min). Additionally, given the ZrP-PS excellent selectivity towards cesium ions, this new adsorbent shows great promise in the treatment of radioactively contaminated waters [129].

## 8. Conclusions

Various novel adsorbents (e.g., ion sieves, aluminum-based materials, minerals, composites, resins) have been successfully used in recent years in the separation processes of valuable (e.g., Li, Rb) or hazardous (e.g., radioactive Cs) low-abundant AMs from aqueous solutions, such as brines and wastewater, which may pose a serious threat to the environment and at the same time may be exploited as a secondary source of critical elements. Current research focuses primarily on the development of new materials in order to maximize their adsorption efficiency (e.g., by changing their composition, application of various synthesis methods, and using adsorption materials in different forms), selectivity (e.g., by introducing into adsorbent materials compounds with affinity for specific metal ions), and reduce the costs associated with the production and application of adsorbents (e.g., development of “one pot” synthesis methods, utilizing easily accessible and inexpensive components, development of simple regeneration methods, and designing of reusable materials).

Although a variety of adsorption materials have been developed to separate Li^+^, Rb^+^, and Cs^+^ ions from solutions, they typically enable the recovery or removal of AMs with varying efficiencies. For example, it has been shown that titanium-based ion sieves, which have been widely and successfully used as efficient and environmentally safe lithium ions adsorbents, when modified (e.g., using polymethyl methacrylate (PMMA) polymer microspheres as matrices), can be applied for the effective separation of cesium ions but are not suitable for the recovery of rubidium ions. Some novel, recently developed adsorption materials based on ferrocyanides can be used as ions exchangers for the removal of cesium, rubidium, and lithium ions, but the efficiency of the adsorption processes may vary significantly due to the different affinity of such adsorbents for metal ions (e.g., Cs^+^ > Rb^+^ > Li^+^). Because of the number of factors (and their mutual dependencies) influencing the adsorption processes (e.g., adsorbent properties, qualitative and quantitative composition of the aqueous solution, properties of separated metal ions), it is difficult to clearly determine which adsorbent is the best in terms of separating a specific metal ion from a specific solution, and in any case the adsorbent performance must be verified experimentally.

However, it should be emphasized that determining the optimal process conditions is usually time-consuming and requires a deeper understanding of the reactions taking place during separation. Hence, one of the trends observed in recent years is the utilization of computational chemistry methods (e.g., DFT) to explain the mechanisms of adsorption processes, which allow for faster acquisition of the necessary information, comparison of theoretical and experimental results, and, consequently, easier determination of the best adsorption parameters.

Particular attention is also paid to the impact of the developed adsorbents on the natural environment and sustainable development. Although the process of separating lithium, rubidium, and cesium ions from brines, sewage, or waste leachates is beneficial for the environment (it helps to limit the extraction of natural ores, reduces the amount of waste, and enables the recycling of raw materials), efforts are being made to develop adsorbents consisting exclusively/mainly of neutral substances. For example, recently, novel adsorbents containing natural zeolites or clays are of great interest. Another solution related to the production of eco-friendly AMs adsorbents, which is part of the so-called “green” chemistry trend, is the utilization of components derived from various types of waste (e.g., biomass carbon derived from citrus peel or aluminosilicate residues obtained from lithium production processing of ores).

In summary, despite the many advantages of the adsorption materials described in this work, there will be a continued search for new adsorbents that will also enable effective separation of AMs on a larger scale, characterized by high efficiency in subsequent cycles and inexpensive. It can be assumed that the interest in complex composite adsorbents based on natural raw materials, which may also contain a number of other components (e.g., MOFs, polymers, plasticizers), will continue due to the possibility of giving them the desired properties (e.g., pore size, surface area, pores regularity, stability) in order to improve their effectiveness under specific conditions and consequently increase their performance. An important issue related to the wider use of various adsorption materials is the possibility of their “regeneration” and multiple applications; hence, many studies concern the development of magnetically recyclable adsorbents. Solving the problems related to the recycling of adsorption materials may have a significant impact on their wider use in the future.

## Figures and Tables

**Table 1:** Examples of the titanium-based ion sieves developed in 2024 for the recovery of lithium ions from various solutions.

Type of Adsorbent/Reference	Type of Solution	Main Advantages
Titanium-based graphene oxide lithium ion sieve HTO@GO [47]	Salt lake brine	The Li^+^ adsorption capacity of HTO@GO was 38.3 mg/g. The results indicate that HTO@GO has good industrial potential with fast Li^+^ adsorption.
Superhydrophilic spinel-type H_4_Ti_5_O_12_ ion sieve with surface wettability adjusted with dual surfactants [48]	Lithium containing solutions	The Li^+^ adsorption was about 85% within 0.5 h, the maximum adsorption capacity was 57.90 mg/g. Adsorption ratio of the ion sieve remained at around 97% after five adsorption–desorption cycles.
The rich-porous HTO with an N-modified interface [49]	Low concentration lithium solutions	The Li^+^ adsorption capacity was 49.05 mg/g, it remained at about 96% after 5 cycles. This method can be good strategy for effective extraction of lithium ions from low concentration solutions.
Iron-doped titanium-based lithium ion sieve (HFTO) [50]	Lithium containing solutions	The Li^+^ adsorption capacity was 34.27 mg/g, after five cycles of adsorption–desorption, adsorbent maintained an adsorption capacity of about 32.00 mg/g.
Highly hydrophilic HTO lithium ion sieve with neodymium doping (1%), Nd-HTO-1% and unmodified HTO ion sieve [51]	Qarhan salt lake brine	The Li^+^ adsorption capacity of Nd-HTO-1% was 43.02 mg/g, Nd-doped HTO increased adsorption capacity by 12.5% compared to the undoped one, and reduced adsorption time by half to 4 h.
HTO [52]	Carbonate-type, sulfate-type, and chloride-type salt lakes solutions	The activity of Li^+^ adsorption sites was affected by the specific hydrochemical types of salt lakes, the adsorption of Li^+^ by HTO was in the order of SO_4_^2−^ (40.08 mg/g) > Cl^−^ (36.66 mg/g) > CO_3_^2−^ (30.18 mg/g).
Hybrid binder (cellulose acetate/sulfonated poly(ether ketone)/poly(vinyl chloride)) granulated HTO [53]	Salt lake brine	Adsorbent showed high extraction capacity (26.54 mg/g), recovery kinetics (19.02 mg/g within 4 h), and dynamic cycling stability (adsorption capacity retention of 94% after 40 cycles).
Porous polyvinyl alcohol/polyacrylamide hydrogels loaded with HTO (HTO-PVA/PAAm hydrogel) [54]	Lithium containing solutions	The HTO-PVA/PAAm adsorption capacity of Li^+^ was 22.16 and 31.31 mg/g in pH 7.2 and 12, the hydrogel is non-toxic and environmentally friendly, potentially can be used in the extraction of Li^+^ ions from salt-lake brine and seawater.

**Table 2:** Examples of the aluminum-based adsorbents used for the recovery of lithium ions from various solutions, developed in 2023–2024.

Type of Adsorbent/Reference	Type of Solution	Main Advantages
Granulated adsorbent HMAG prepared using Li/Al-LDH powder, PVC/PM as the binder and *N*, *N*-dimethylformamide as bonding agent [74]	Qarhan low grade salt lake brines	HMAG granules exhibited higher than the conventional adsorbent granules powder loading (86%) and superior hydrophilicity, demonstrated a remarkable adsorption performance (Li^+^ adsorption capacity was 2791.00 mg/L after a 6 h feed period).
Granular aluminum-based adsorbents with polysulfone (PSF) as a binder regulating the structure, and poly(ethylene glycol) (PEG) and polyvinylpyrrolidone (PVP) as pore-making agents [75]	Old brine of Qarhan salt lake	High adsorption capacity of optimized PSF-PEG containing adsorbent (99.02% after 10 static adsorption–desorption cycles), excellent cyclic stability, adsorbent with adjustable pore structure and surface properties.
Co-LDHs-SO_4_ [76]	Sulfate-type West Taijinar salt lake brines	Adsorbent characterized by high anti-deactivation property, excellent structure reversibility, high adsorption capacity (<10 mg/g), excellent Li^+^/Na^+^, Li^+^/Mg^2+^, Li^+^/K^+^ separation coefficients (236.7, 187.2 and 282.6, respectively).
Aluminum-based H-LDHs prepared by a precipitation method followed by water elution [77]	Model solutions	High adsorption capacity of adsorbent (8.4 mg/g of Li^+^), unique self-healing ability and cycling stability.
Granulated LDHs adsorbents produced by novel extrusion granulation method and with an antisolvent strategy [78]	Ultrahigh Mg^2+^/Li^+^ salt lake brines	Great adsorption performance of adsorbent granules in brine with a Mg^2+^/Li^+^ mass ratio of 294.24, Li^+^ adsorption capacity was stable at 4.45–4.86 mg/g in 24 cycles without structural transformation of material.
Li/Al-LDHs, with an interlayer restoration strategy for SO_4_^2−^ intercalated Li/Al-LDHs [79]	Sulfate-type brines	The cyclic Li^+^ adsorption and desorption capacities in the enhanced process reached higher values in comparison to conventional process.
Magnetic aluminum-based adsorbents (MLDHs) prepared by homogeneously doping silicon dioxide coated ferric tetroxide nanoparticles (Fe_3_O_4_@SiO_2_) into LDHs [80]	Aqueous solutions, mixed solution of various metal ions	Adsorption capacity of MLDHs for Li^+^ reached 8.22 mg/g, MLDHs have good selectivity for lithium ions and exhibited good stability.

**Table 3:** Examples of the complex and composite adsorbents used for the recovery of cesium, lithium, and rubidium ions from various solutions, developed in 2023–2024.

Type of Adsorbent/Reference	Type of Solution	Main Advantages
Porous fiber-supported metal tin sulfide PVC-[Me_2_NH_2_](2)Sn_3_S_7_ [111]	Simulated wastewater with cesium ions	High adsorption capacity (419 mg/g) in 30 min and a wide active pH range. After 50 cycles of regeneration, the adsorbent still had good adsorption performance.
Cation (Na^+^ or NH_4_^+^) intercalated lamellar MoS_2_ [112]	Simulated brines with Cs^+^, Li^+^, Na^+^, K^+^, Mg^2+^, Ca^2+^ ions	The best-performing NH_4_^+^-intercalated material was highly selective for cesium ions over competing ions.
Ion-imprinted adsorbent Cs_2.33_Ga_2.33_Sn_1_·_67S_8·H_2_O [113]	Actual ^137^Cs-liquid-wastes generated during industrial production	The adsorbent reached adsorption equilibrium for Cs^+^ within 5 min (adsorption capacity of 246.6 mg/g), enabling highly selective removal (over 99%) of cesium ions from complex wastewater.
pNCE-SS@UiO-66 [117]	Synthetic brines with Li^+^ and Mg^2+^ ions	High selectivity towards Li^+^ ions with high adsorption capacity (1.47 mmol/g), easy regeneration of adsorbent in warm water.
Mesoporous spherical Zn(4-hzba)MOF [119]	Model solution with Sr^2+^, Cs^+^, Na^+^, K^+^, Ca^2+^, Mg^2+^	High selectivity and adsorption efficiency of Sr^2+^ and Cs^+^ ions in simulated seawater (92.3% and 96.7%, respectively).
KCoFC@ZIF [122]	Seawater	KCoFC@ZIF showed 8-folds higher rubidium ions uptake compared to KCoFC and was efficient for selective Rb^+^ ions uptake in seawater.

## Data Availability

Not applicable.
